# CFM-YOLOv5:CFPNet moudle and muti-target prediction head incorporating YOLOv5 for metal surface defect detection

**DOI:** 10.1371/journal.pone.0289179

**Published:** 2023-12-07

**Authors:** Yuntao Xu, Peigang Jiao, Jiaqi LIU

**Affiliations:** School of Engineering Mechanical, Shandong Jiaotong University, Jinan, Shandong Province, China; Hainan Normal University, CHINA

## Abstract

Aiming at the problem of low efficiency of manual detection in the field of metal surface defect detection, a deep learning defect detection method based on improved YOLOv5 algorithm is proposed. Firstly, in the feature enhancement part, we replace the multi-head self-attention module of the standard transformer encoder with the EVC module to improve the feature extraction ability. Second, in the prediction part, adding a small target detection head can reduce the negative impact of drastic object scale changes and improve the accuracy and stability of detection. Finally, the performance of the algorithm is verified by ablation experiments and analogy experiments. The experimental results show that the improved algorithm has greatly improved mAP and FPS on the data set, and can quickly and accurately identify the types of metal surface defects, which has reference significance for practical industrial applications.

## Introduction

With the development of artificial intelligence, more and more industries integrate enterprise technology with artificial intelligence, such as Tesla. These phenomena reflect that artificial intelligence has gradually become an indispensable part of the industrial field and has gradually become one of the world’s hot industries. In the industrial manufacturing industry, metal workpieces are an important part of some products. The quality of metal workpieces not only affects the life of products and the development of enterprises, but also involves the safety of users. Therefore, adopting accurate and fast target detection algorithms plays an important role in industrial manufacturing.

In recent years, with the development of deep learning, more and more studies have used convolutional neural networks and other deep learning methods for metal defect detection. These methods can automatically learn feature representation and perform well on large-scale data sets. However, metal defect detection faces some major problems. First of all, since metal defects are usually related to materials and construction techniques, there is a problem of class imbalance, that is, the number of normal samples far exceeds the number of defective samples. This may lead to the model biased towards normal samples and insufficient detection performance for defect samples. Secondly, metal defects are often small, even small subtle changes, which poses a challenge to traditional defect detection methods. Small target detection requires attention to details, context information, and the processing power of high-resolution images. Finally, there are many types of metal defects, such as cracks, holes, scratches, etc. Different types of defects may require different detection methods and models. Therefore, how to effectively detect and classify different types of defects is still a challenge.

There are many problems in the traditional metal workpiece surface defect detection algorithms, such as SIF and THOG algorithms [1, 2], including cumbersome processes, difficulty in achieving automated detection, slow detection speed, low efficiency, and prone to false detection and missed detection. In contrast, the object surface defect detection method based on deep learning provides an end-to-end solution. This method uses convolutional neural network to learn defect features autonomously, which can express and understand defect information more accurately, so as to achieve more accurate detection results. At present, the widely used deep learning target detection algorithms are mainly divided into two categories, one of which is the two-stage target detection algorithm. RCNN is a pioneering work in the field of target detection. It extracts candidate regions by selective search algorithm, then adjusts these regions to a fixed size, extracts features by convolutional neural network (CNN), and uses support vector machine (SVM) to classify each region [3]. Fast-RCNN is an improved algorithm of R-CNN, and its main goal is to improve the detection speed. It introduces the ROI pooling layer, obtains the feature map through a CNN forward propagation, and uses the ROI pooling layer to extract the features of each candidate region. In addition, Fast-RCNN combines the SVM classifier and the bounding box regressor into a multi-task loss function to achieve end-to-end training [4]. Faster-RCNN is a further improvement of Fast-RCNN. It replaces selective search with RPN (Region Proposal Network) and shares feature extraction network with Fast-RCNN, which reduces the amount of calculation and greatly improves the detection speed [5]. The other is a single-stage target detection algorithm. The detection of the target is regarded as a regression problem represented by the YOLO series algorithm [6]. There is no need to generate candidate boxes, and the location and category information of the target are directly predicted through the network. Therefore, the single-stage target detection algorithm can save a lot of computing time.

Feature pyramid is an important part of object detection, semantic segmentation, behavior recognition and so on, which has a very good performance in improving the performance of the model [7, 8]. There are two ways to construct the pyramid. One is to generate layers with different resolutions by multiple downsampling. This method is widely used. The more common application is the SSD algorithm. In metal defect detection, the algorithm can be used to detect defects of various sizes, such as cracks, holes, etc. However, when dealing with small targets, attention should be paid to details and context information, as well as appropriate image resolution to improve defect detection performance [9]. The FPN algorithm performs well in multi-scale feature fusion and context information utilization, but its generalization ability may be limited by specific defect types. Different types of metal defects may require additional model adjustments and data training to obtain better detection results [10]. Secondly, it is constructed by multiple branches of dilated convolution with different cavity rates. At present, ASPP algorithm is applied in this field. By using dilated convolution with different expansion rates, features with different scale receptive fields can be obtained. This is very useful for multi-scale defects in metal defect detection, which can better capture defect information of different sizes. Since ASPP introduces multiple parallel branches, the number of parameters and the amount of calculation of the network will increase. This may lead to large memory usage and increase the computational cost of model training and inference [11].

In the process of metal forging, there will be different degrees of damage to the metal surface, such as cracks, scratches, depressions, etc. When these damages exist in the same image, there will inevitably be more background interference and a variety of indistinguishable defect categories. Using traditional target detection algorithms and deep learning algorithms to detect will undoubtedly increase the difficulty.

Based on the above analysis, this paper proposes a CFM-YOLOv5 detection algorithm for metal surface defect detection based on YOLOv5. The algorithm is mainly to solve the background clutter in the industrial scene. The diversity of defect scales and the large number of small defects lead to the inability to effectively detect metal surface defects. The model integrates the Explicit Visual Center (EVC) module, which is mainly composed of two parallel connected blocks, in which lightweight MLP is used to capture global information. At the same time, in order to retain local information, a learnable visual center mechanism (LVC) is used to aggregate local region features in the layer to improve feature generalization and robustness. On the basis of the original three detection heads, a small object detection head is added. The four-head structure can alleviate the negative impact of drastic target scale changes, and adopt the Mosica data enhancement method to enrich the data set while reducing the GPU’s existing. Through the model training and ablation experiment of NEU-DET data set, more convincing data can be obtained. The main contributions of this paper are as follows:

1. Replace the multi-head self-attention module of YOLOv5’s standard Transformer encoder with a lightweight MLP layer, and adopt a parallel learnable visual center mechanism to aggregate the local key areas of the input image.
2. based on the three prediction heads of the original YOLOv5, a small target detection head is added to eliminate the negative effects of defects and increase the detection performance and robustness of small targets.
3. CFM-YOLOv5 achieves a large performance gain in metal surface defect detection.

This paper proposes CFM-YOLOv5 for metal surface defect detection. This method uses some technical combinations in the deep learning algorithm, such as replacing the Transformer encoder block, increasing the attention head, data set expansion and enhancement methods. The effectiveness of the algorithm is verified by ablation experiments. The experimental results show that the model can achieve high detection accuracy while having real detection ability. The ablation experimental results show that the mAP of CFM-YOLOv5 is 78.6:%, which is about 4.4:% higher than that of the benchmark YOLOv5 model. It can solve the complex problems such as strong background interference, large change of defect scale, large number of small defects and easy confusion of defect targets in industrial scenes. It can effectively deal with different types of defect detection tasks while enhancing robustness and accuracy, and provide comprehensive detection capabilities.

## Related work

### Common target detection

Deep learning is a new research direction in the field of machine learning. It is introduced into machine learning to realize artificial intelligence faster. With the development of technology, people have been trying to make the machine realize autonomous learning, and autonomously detect the task target after actively learning a lot of basic knowledge. Although this is a simple task for humans, it is very difficult for computers to recognize objects.

In recent years, traditional deep learning has made remarkable progress in the field of metal surface defect detection. Relevant research focuses on how to use deep learning methods to improve the accuracy and efficiency of metal surface defect detection. Among them, some important research work includes a hybrid deep learning method proposed by Dong et al., which combines models such as CNN and GAN to improve detection performance and positioning accuracy of defect areas [12]. Zheng et al.’s research focused on building a high-performance CNN model and improving the robustness of the model through data augmentation technology [13]. Zeng et al.used deep convolutional neural network (CNN) to build a model to classify and locate different types of metal defects [14]. Liu et al.proposed a CNN-based method for the detection and segmentation of metal plate surface defects [15]. These researches focus on constructing high-performance deep learning models, feature extraction, defect classification and localization, and provide useful methods and techniques for metal surface defect detection.

In recent years, in addition to the research on traditional deep learning algorithms, researchers have proposed many innovative deep learning methods in the field of metal surface defect detection. Yang et al.proposed a metal surface defect detection method based on Squeeze-and-Excitation network, which improves the accuracy and robustness of the detection by enhancing the network’s ability to model key features [16]. Luo et al.proposed an improved deep learning method, which improved the accuracy and robustness of metal surface defect detection by introducing SE attention mechanism and multi-scale feature fusion [17]. Sun et al.studied defect detection based on deep convolutional neural network (CNN) model and multi-layer pooling convolution and introduced image enhancement and sample balance techniques [18]. Their research focuses on the design of multi-scale CNN models, and uses image enhancement and sample balance techniques to improve the accuracy and robustness of detection.

In the past five years, researchers have continuously improved the application effect of deep learning in metal surface defect detection by improving network structure, introducing attention mechanism and multi-scale feature fusion. These research results highlight key research priorities such as improving accuracy, robustness, and adaptability. It should be noted that specific research methods and priorities may vary according to different purposes and methods.

### Transformer

Transformer is a revolutionary deep learning model that was originally applied to natural language processing tasks such as machine translation and language modeling [19]. Compared with the traditional sequence models (RNN and CNN), the Transformer model adopts a new way to process sequence data, that is, the attention mechanism is used to learn the relationship in the sequence. In the Transformer model, the self-attention mechanism is a technique for calculating the correlation between sequence elements. Each element is associated with all other elements in the sequence and the correlation weight with other elements is calculated. This parallel processing method makes the Transformer model not need to process each element in turn according to the traditional method, so it has higher efficiency and performance in processing long sequence data.

In addition to the self-attention mechanism, the Transformer model also uses a new residual connection and normalization technique to accelerate the model and prevent gradient disappearance during training. As these innovative technologies have brought significant performance improvements, Transformer model has become one of the important technologies in the field of natural language processing, and has achieved remarkable results in machine translation, text generation, sentiment analysis and other fields.

In YOLOv5, Transformer is used to encode and decode feature maps to improve detection accuracy and speed. In summary, the Transformer module includes multi-head self-attention mechanism, feedforward neural network and residual connection, which can effectively capture the spatial and semantic information in the feature map.

### MLP

Multilayer perceptron (MLP) is a common artificial neural network model, which has been widely studied, including model structure, training algorithm and application field [20, 21]. Researchers have been exploring how to design more efficient and accurate MLP network structures, such as convolutional neural network (CNN) and recurrent neural network (RNN). The training algorithm of MLP is also an important research direction, such as back propagation algorithm (BP) and stochastic gradient descent (SGD), and some optimization algorithms based on adaptive learning rate such as Adam and Adagrad. In the field of application, MLP has been widely used in image recognition, natural language processing, speech recognition and recommendation systems. Researchers are also constantly exploring the application of MLP in new application fields [22]. At the same time, in YOLOv5, MLP is used to process input feature vectors in order to better identify targets [23]. It can perform nonlinear modeling, process high-dimensional data, perform end-to-end learning, perform deep learning and transfer learning, and has many advantages. In general, the research of MLP is deepening, and there are still many directions worth exploring in the future.

## Proposed methods

In this chapter, the CFM-YOLOv5 algorithm for metal defect detection will be further elaborated. The CFM-YOLOv5 proposed in this paper is based on the v5 s model in the YOLOv5 series. The model performs best on devices with limited computing resources, such as mobile devices or edge devices. Its detection speed is the fastest, and it is integrated into the EVC module and adds a small target detection head. YOLOv5 was created shortly after v4 was proposed, which has insufficient innovation. Compared with Faster R-CNN and RetinaNet, the accuracy of YOLOv5 is slightly lower, and the detection effect of small targets is not satisfactory. The method proposed in this paper can make up for the defects and apply to more industrial scenarios.

### CFM-YOLOv5

There are five types of YOLOv5 models, including v5s, v5m, v5l, v5x, and v5n. Among them, 5s has the fastest speed, but the accuracy is low. The speed and accuracy of v5m are at a balance point. The accuracy of v5l is the highest, but the speed is slow. The speed and accuracy of v5x are very high, but more computing resources are needed. Because this paper is mainly suitable for industrial detection scenarios and has high real-time requirements, this paper takes YOLOv5s as the baseline for research.

**Fig 1:**
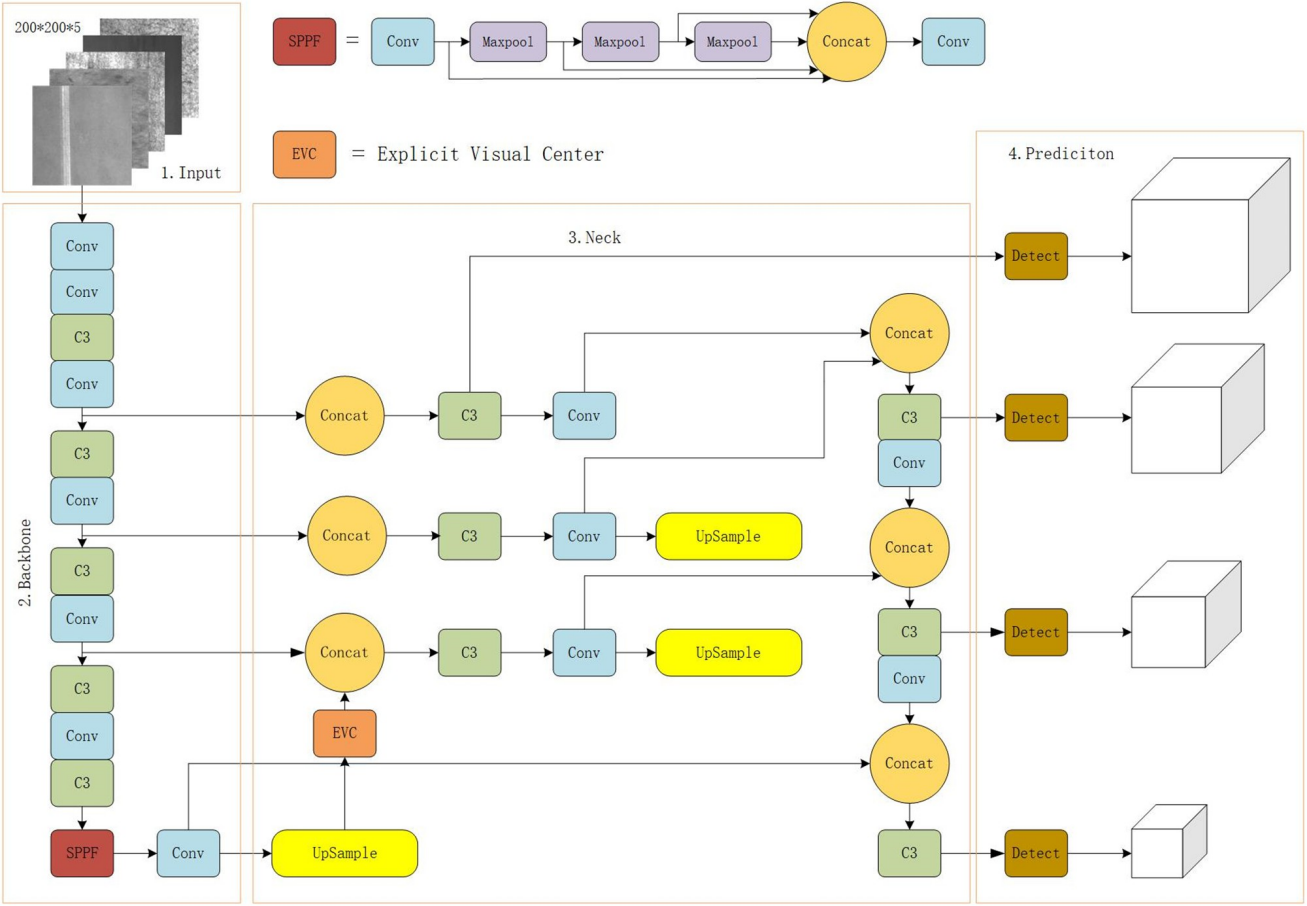
CFM-YOLOv5 overall structure diagram. (1) Backbone uses YOLOv5s-6.0 version. (2) The Neck section adds an EVC module to replace the Transformer. (3) The Predicition module uses four detection heads.

The overall diagram of MSFT-YOLO is shown in Fig 1, which is mainly composed of four parts: input, backbone, feature enhancement and prediction. In the backbone section, we are based on version 6.0. In the feature enhancement part, we replace the multi-head self-attention module of the standard transformer encoder with the MLP module. Compared with the traditional transformer encoder head, the MLP architecture we adopt is not only simple in structure, but also has lighter volume and higher computational efficiency. At the same time, a visual center mechanism is added along the lightweight MLP to aggregate the local angular regions of the input image. As mentioned above, MLP and visual center mechanism are called EVC module. In this paper, there are small targets in the NEU-DET defect data set, and it is very difficult to detect them using traditional v5s. Because small targets usually have special shapes and smaller sizes, as well as lower contrast and complex backgrounds, they are difficult to mark or identify. For this reason, a detection head for predicting small targets is added to the prediction part, and the original three detection heads are increased to four. While detecting training samples, smaller samples will not be ignored, and the overall detection accuracy and stability are greatly increased.

By observing the NEU-DET defect data set, it is not difficult to find that the defect samples are characterized by a large number of defects and large background differences. Due to the industrial production environment, the specifications of different defect types vary greatly. The CFM-YOLOv5 model proposed by us is developed based on YOLOv5s. In the feature enhancement part and the prediction part, the EVC module and the small target detection head are combined to enrich the detection process and achieve the best detection results.

### EVC

**Fig 2:**
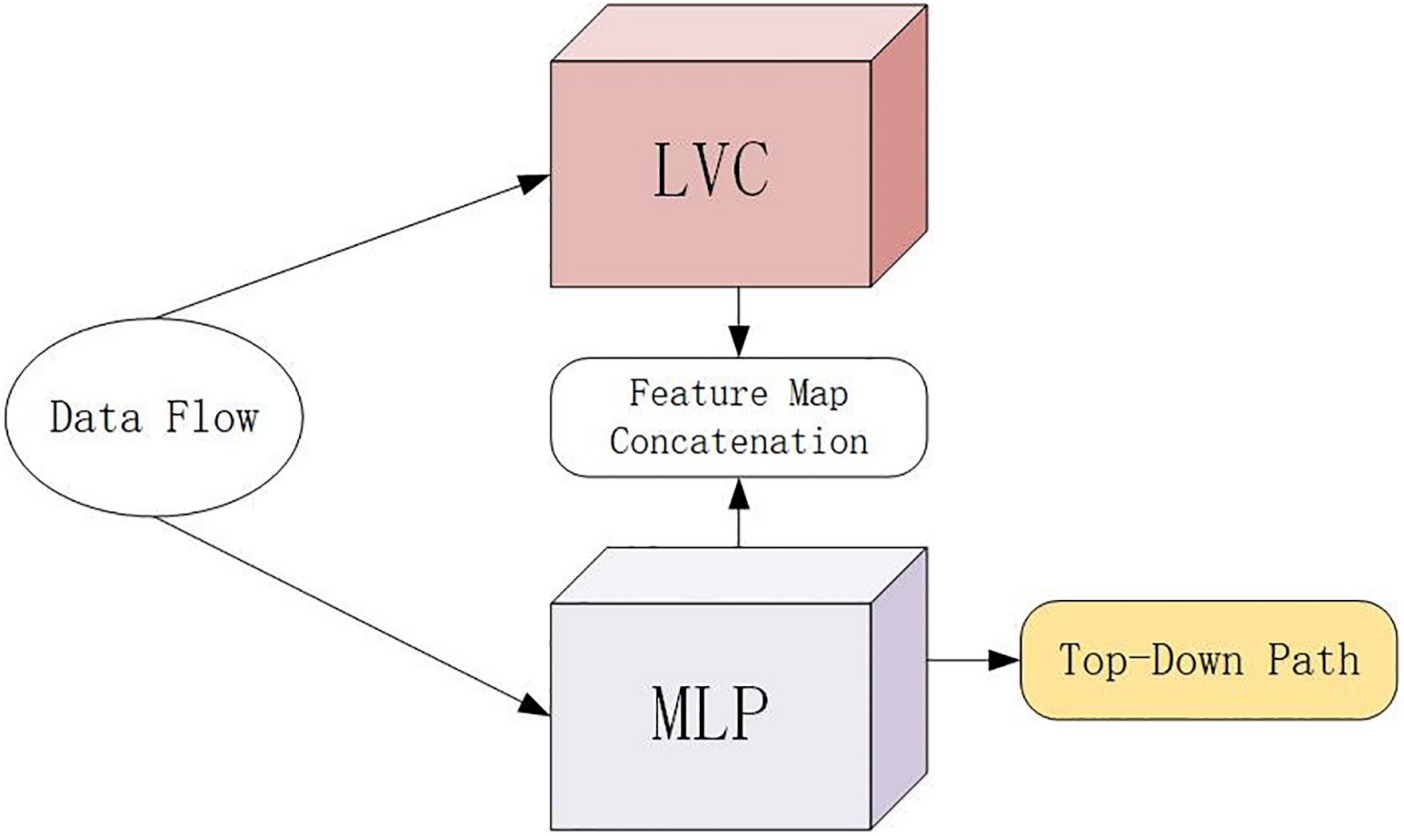
EVC module structure diagram.

In this paper, the EVC module is used to replace the multi-head self-attention module of the standard transformer encoder. This part is mainly composed of two parallel connected blocks, in which the lightweight MLP is used to capture the global information. At the same time, in order to retain the local information, the learnable visual center mechanism (LVC) is used to aggregate the local area features in the layer. The structure is shown in Fig 2.

The result feature maps of these two blocks are connected together along the channel dimension as the output of EVC for downstream identification. The implementation process can be realized by the following formula:

$$\begin{array}{c} X=Cat(MLP\left(X_{in}\right);LVC\left(X_{in}\right)) \end{array}$$ (0.1)

X is the output of EVC. Cat()represents the cascade of feature maps along the channel dimension. *EVC*(*X*_*in*_) and *LVC*(*X*_*in*_) represent the output features of the MLP and the learnable visual center mechanism, respectively.

The MLP used in EVC is mainly composed of deep convolution module [24] and channel MLP module, and the input based on MLP module is the output of deep convolution module [25]. These two blocks are then operated by DropPath [23] and channel scaling [26] to improve feature generalization and robustness. LVC is an encoder with an inherent dictionary, and has two components and an inherent codebook and a set of scaling factors [27].

EVC is a generalized intra-layer feature adjustment method. It can not only extract the global long-distance correlation, but also retain the local information of the input image to the greatest extent, which is very important for the large-scale defect data set. However, using EVC at each level of the feature pyramid will lead to large computational overhead, so this paper adds an EVC module.

### Prediction head for tiny objects

By studying the NEU-DET data set, it is found that the target specifications on the defect image category are small, and only a small pixel area is occupied in the image. The traditional detection algorithm may not detect these targets accurately. Therefore, we add a prediction head for small target detection. The four-head structure composed of the other three prediction heads can reduce the negative impact of drastic object scale changes and improve the accuracy and stability of detection. At the same time, this can also make YOLOv5 play a better role in a wider range of application scenarios and improve its practicability and practical value. As shown in Fig 1, the predicted head we added is generated from low-level, high-resolution feature maps and is more sensitive to small objects [28, 29].

## Experimental results and discussion

**Fig 3:**
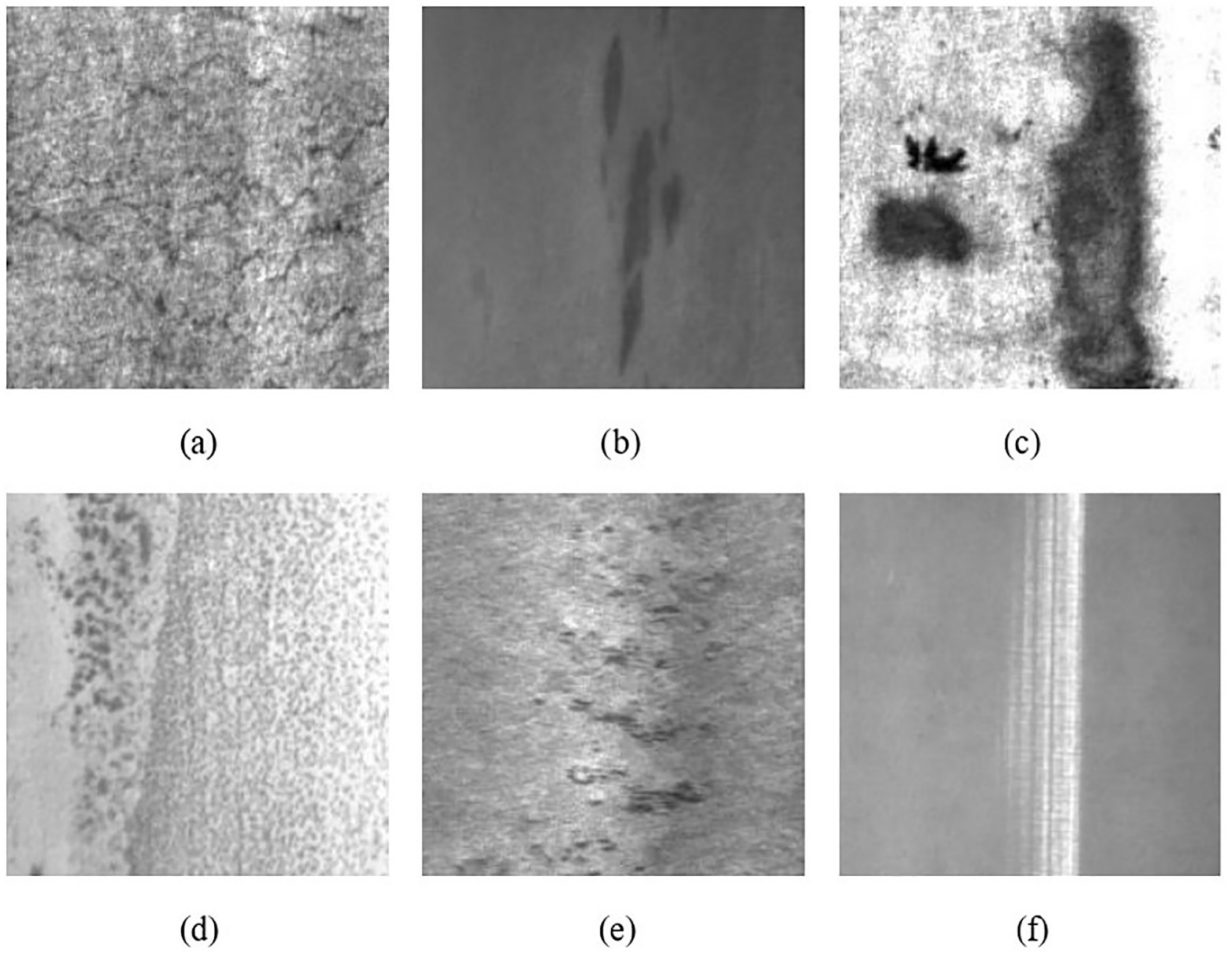
Six types of metal surface defects. (a) crazing, (b) inclusion, (c) patches, (d) pitted surface, (e) rolled-in scale, (f) scratches.

In this paper, the NEU-DET defect data set released by Northeastern University is used as the training sample. The data set contains six types of metal surface defects such as crazing, patches, and pitted surface. Each type has 300 grayscale images, a total of 1800 grayscale images. Each image contains a variety of defects. The image is shown in Fig 3.

### Pre-experiment preparation

#### Experimental environment

Our CFM-YOLOv5 model is implemented on the Windows 10 platform using the PyTorch framework. The Nvidia GeForce RTX 3060 graphics card is used for computing,and the video memory size is 12G. The device uses Intel(R)Xeon(R)W-3225 CPU @ 3.70GHz. The CUDA version is 11.1, the CUDNN version is 8.0, and the Python version is 3.9. The batch size was chosen as 16. We updated the network for 100 epochs. The optimizer was chosen as Adam with a learning rate as 10.

#### Mosica enhancement

In order to give full play to the feature extraction, detection and classification capabilities of deep learning networks, it is very important to train them on large-scale data sets. However, the NEU-DET data set used in this study is relatively small. Therefore, data augmentation is required before model training to achieve satisfactory training results. Data augmentation is a technique commonly used in machine learning and deep learning. It creates more and more diverse training samples by a series of transformations and expansions of the original data. The commonly used geometric transformation methods are: flip, rotate, cut, zoom, pan, jitter, etc. The commonly used pixel transformation methods are: adding salt and pepper noise, Gaussian noise, Gaussian blur, adjusting HSV contrast, adjusting brightness, saturation, histogram equalization, adjusting white balance, etc. The commonly used methods for detecting classification tasks are: Mixup, Cutout, Cutmix, Mosaic, etc. Among them, Cutout and Cutmix are the differences in pixel values in the filled area. Mixup and Cutmix are the differences in the way of mixing two samples. Mosaic’s idea is to randomly crop four pictures and then splice them into one picture as training data [30].

In order to further clarify the data enhancement effect and the enhancement method in line with the defect detection in this paper, the experiment is based on the NEU-DET defect data set, and the traditional enhancement method is compared with Mixup, Cutout, Cutmix, and Mosaic enhancement methods. The experimental results are shown in Table 1.

**Table 1:** Comparison of different data enhancement experiments.

Image	Salt	Cutout	Cutmix	Mixup	Mosaic
NEU-DET Cls(:%)	76.5	77.1	78.6	77.4	79.2
NEU-DET Loc(:%)	46.3	46.7	47.3	45.8	49.2
Pascal NEU-DET Det(mAP)	68.5	71.2	74.3	75.5	76.2

Based on the data in Table 1, Cls (Classification), Loc (Localization) and mAP of NEU-DET are used as indicators to measure the effect of data enhancement. Combining the results of the three indicators, Mosaic has the highest values of Cls and Loc in several data enhancement methods, and the key value mAP to measure the detection accuracy is the highest. Therefore, this paper uses the Mosaic method as the core method of data enhancement.

**Fig 4:**
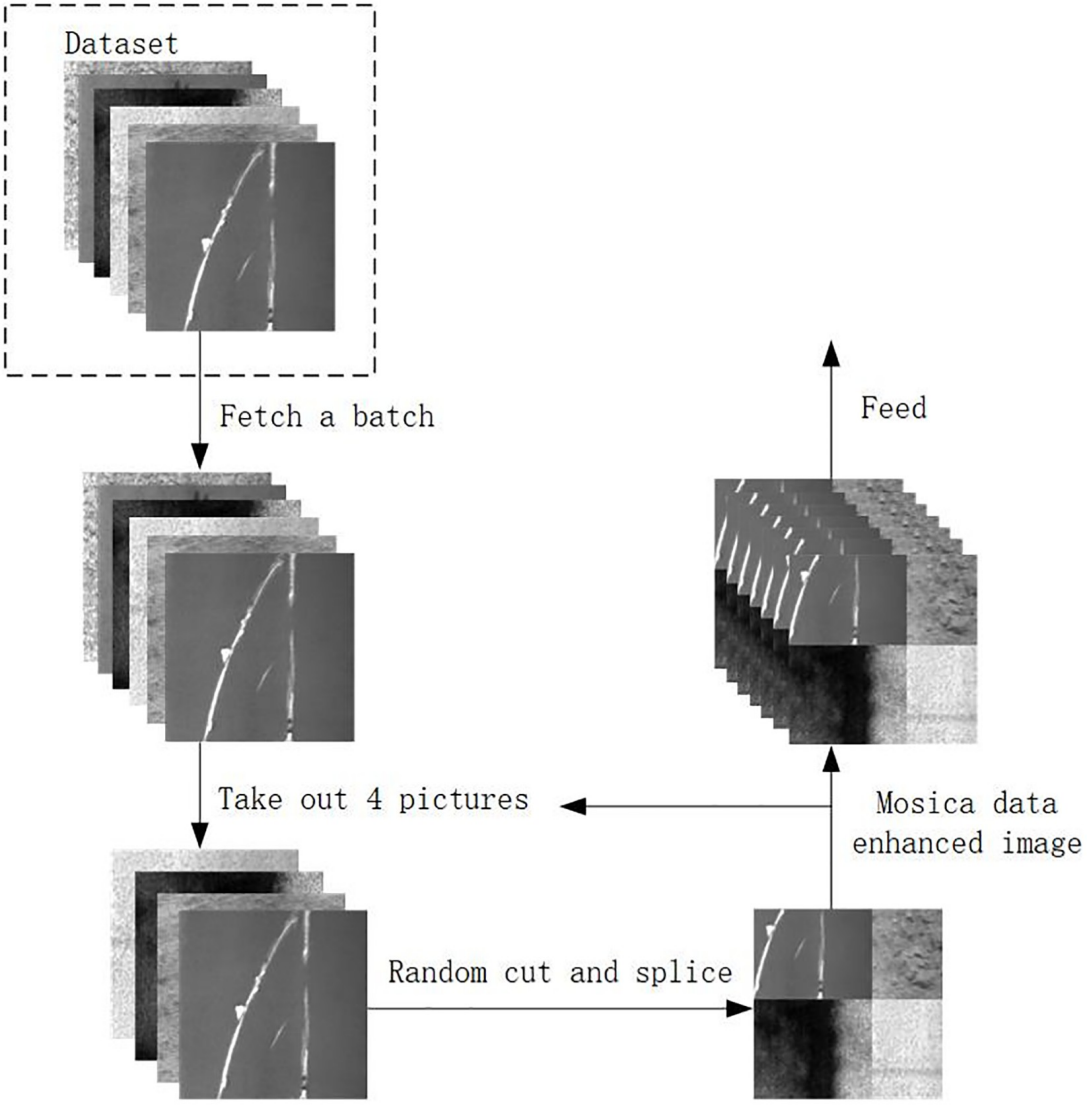
The mosica data enhancement schematic diagram.

As shown in Fig 4, the Mosaic augmentation process, involves the following steps: First, four randomly selected images from the training dataset are chosen as inputs. These selected images undergo random cropping to generate four distinct cropped regions. Next, the four cropped regions are arranged and combined in a mosaic fashion, maintaining certain proportions. This arrangement results in a synthesized Mosaic image. The coordinates and sizes of the bounding boxes are adjusted based on the dimensions of the synthesized image and the location information of the target objects, ensuring their alignment with the new image. Finally, the adjusted bounding boxes, along with their corresponding class labels, are utilized as training samples [29].

By incorporating Mosaic augmentation, the training dataset is enriched, providing the model with exposure to diverse scenarios, scales, and variations of objects. This augmentation technique effectively enhances the model’s robustness and generalization capability.

### Experimental evaluation indicators

In the field of deep learning defect detection, the evaluation model performance indicators usually use Precision, Recall, Mean Average Precision, FPS, etc. Where Precision (P) is the proportion of the samples in which the model predicts a positive case (positive sample) and the actual positive case. Recall (R) represents the proportion of samples that are actually positive, and the model correctly predicts positive examples. Mean Average Precision (mAP) calculates the average accuracy of different categories and uses their average as the final performance measure. FPS represents the processing speed of the model in image or video processing. The higher the FPS, the faster the model can process images or videos. P, R and mAP are calculated using equations:

$$\begin{array}{c} P=\frac{TP}{TP+FP} \end{array}$$ (0.2)

$$\begin{array}{c} R=\frac{TP}{TP+FN} \end{array}$$ (0.3)

$$\begin{array}{c} mAP=\frac{1}{\left|N\right|}\sum_{i=1}^{n}AP_{i} \end{array}$$ (0.4)

In Eqs (2) and (3), *TP*, *FP* and *FN* are the number of correctly identified positive samples, the positive model that has been misallocated, and the negative samples that have been misallocated, respectively. The *AP* value in formula (4) refers to the area of the P-R curve. The *mAP* is obtained by averaging the *AP*_*s*_ of all categories, and the *AP*_*i*_ represents the average accuracy of the class I target, and n represents the number of categories.

### Experimental results

**Fig 5:**
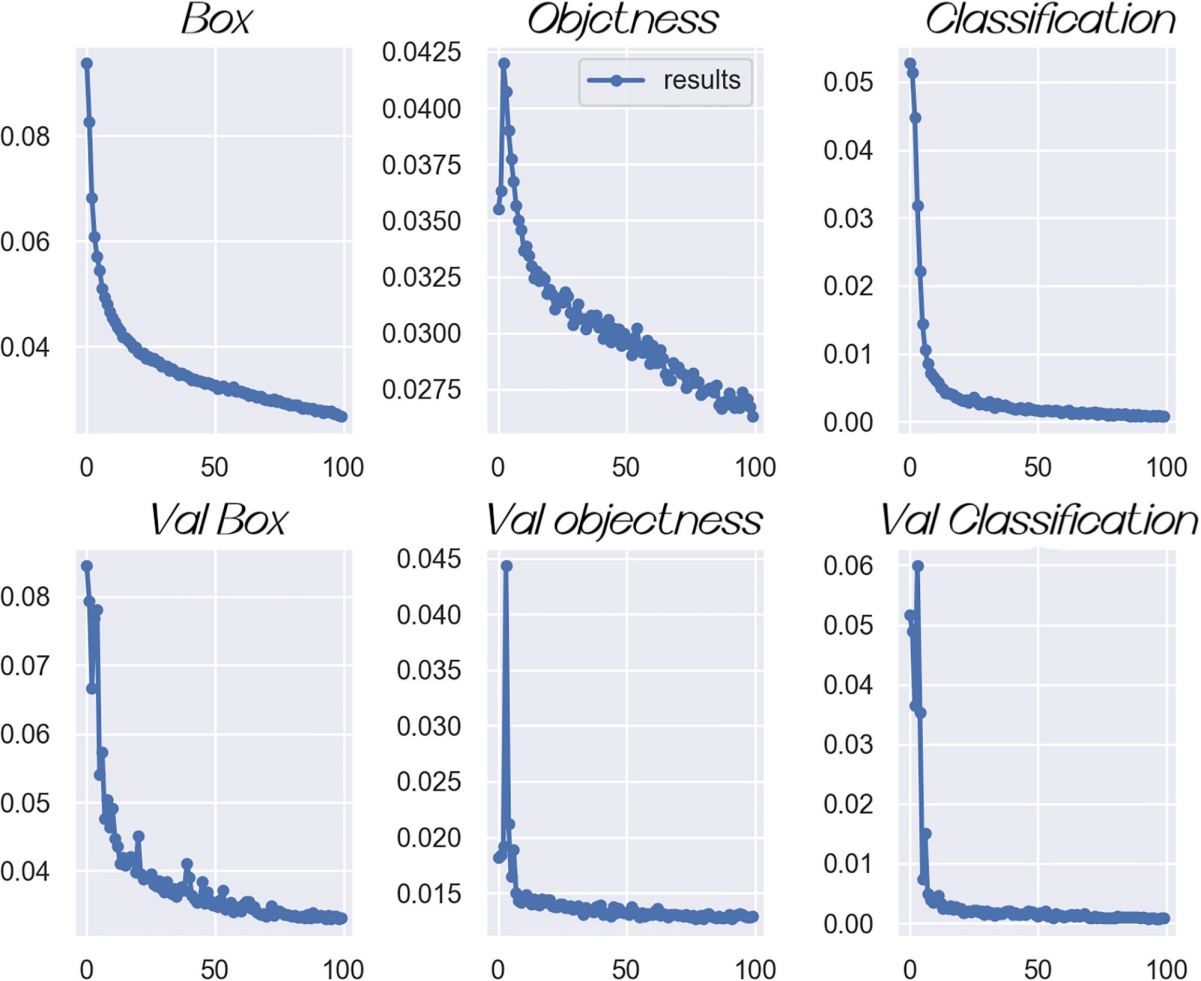
The positioning loss, confidence loss and classification loss data obtained from the experimental training results.

Through the training sample experiment of CFM-YOLOv5, Fig 5 can be obtained, which shows the location loss (box_loss), confidence loss (obj_loss) and classification loss (cls_loss). For these three indicators, when the positioning loss is smaller, the anchor frame grabbed by the target is smaller. When the confidence loss is smaller, the target detection is more accurate. When the classification loss is smaller, the defect target classification is more accurate. The training and verification results can be obtained from Fig 5. The box_loss for target detection and localization is about 0.03, the confidence loss (obj_loss) is less than 0.02, and the classification loss (cls_loss) is close to 0. The experimental results show that the method can accurately detect the target and its position, and can perform effective classification, but there are some errors.

**Fig 6:**
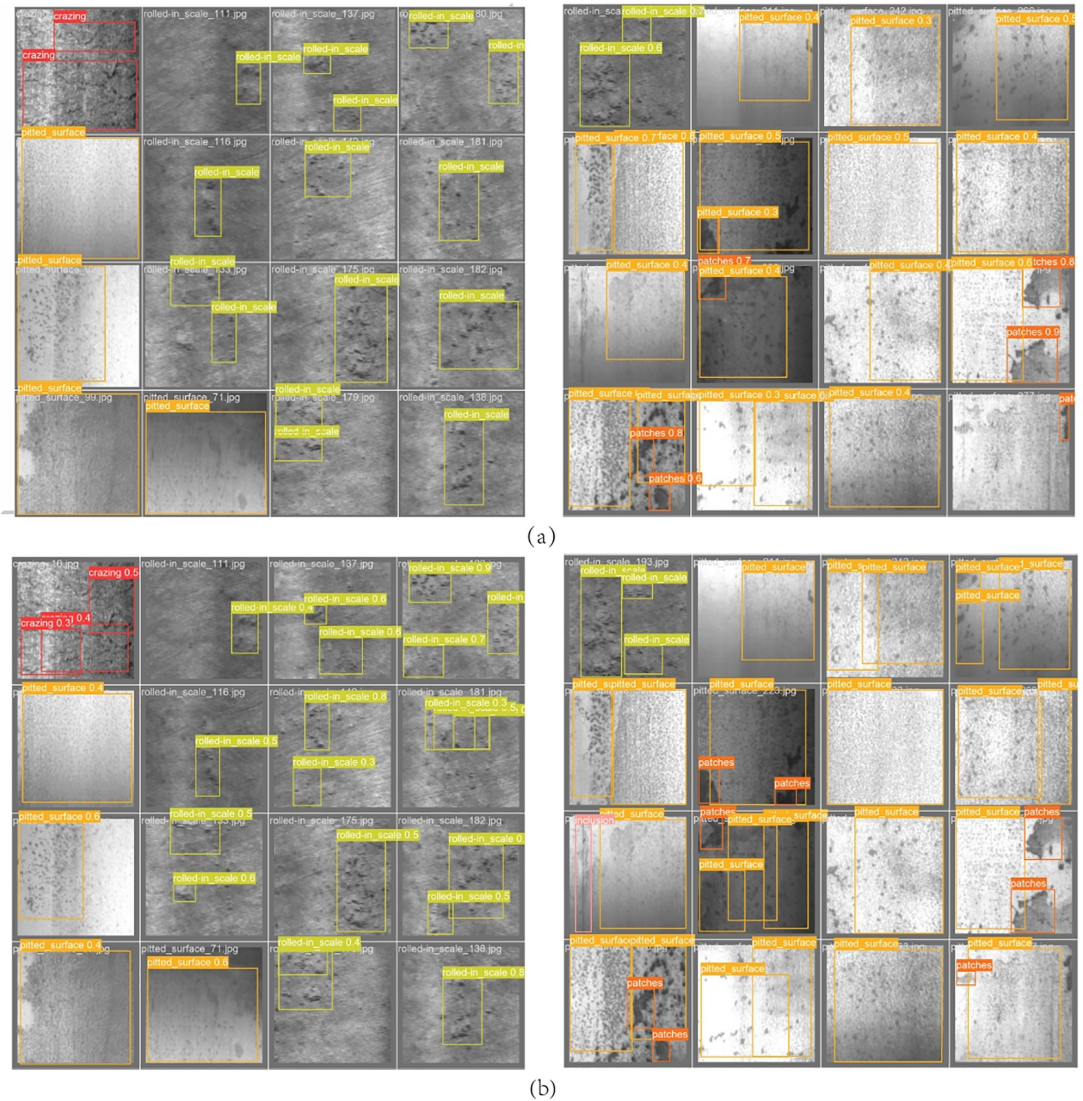
The prediction results of deep learning algorithm for defect data sets. (a) The traditional YOLOv5 prediction box for defect types. (b) CFM-YOLOv5 algorithm for defect type prediction box.

In Fig 6, this paper randomly selects two sets of images from the NEU-DET defect data set, and uses the traditional YOLOv5s algorithm and CFM-YOLOv5 to detect these three images. The prediction results reflect the optimized performance.

The performance of the improved CFM-YOLOv5 model is shown in Table 2, where Precision (P), Recall (R) and Mean Average Precision (mAP) were 0.894, 0.970, 0.786. Moreover, the mAP value of the six defect types can reach up to 0.953. The experimental results show that the improved CFM-YOLOv5 model has high recognition accuracy for metal surface defects.

**Table 2:** Performance of CFM-YOLOv5 model.

Evalution Metrics	Crazing	Inclusion	Patches	Pitted Surface	Rolled-in Scale	Scratches	All
P	0.690	0.854	0.899	0.921	0.812	0.889	0.894
R	0.775	0.965	0.960	0.955	0.900	0.958	0.970
mAP	0.473	0.892	0.953	0.863	0.647	0.885	0.786

### Ablation experiment

As shown in Table 3, it can be observed from the experimental data that the defect detection of Rolled-in Scale and Scratches is greatly improved by replacing the multi-head self-attention module of the standard transformer encoder with the EVC module in the feature enhancement part, and SmallOBJ has more influence on Crazing, Rolled-in Scale and Scratches, which have a markup of more than 1%. Through the analysis of the test results, although the detection speed is reduced by 40%, it still has the potential for real-time detection. The detection accuracy is improved from 0.742 to 0.786, and it is not difficult to see a qualitative leap in accuracy.

**Table 3:** Ablation study on CFM-YOLOv5l.

Method	Crazing	Inclusion	Pathces	Pitted Surface	Rolled-in Scale	Scratches	mAP	FPS
YOLOv5s(basic)	0.403	0.862	0.944	0.832	0.530	0.881	0.742	52.5
YOLOv5s+EVC	0.365	0.886	0.945	0.772	0.652	0.929	0.758	42.1
YOLOv5s+SmallOBJ	0.489	0.856	0.933	0.771	0.666	0.900	0.769	41.2
YOLOv5s+All	0.473	0.892	0.953	0.863	0.647	0.885	0.786	31.2

In order to prove the advantages of CFM-YOLOv5, we compare the proposed method with the existing representative models. ResNet18-dcn, ResNet18-ds and ResNet18-dsf are all improved versions of ResNet18, which are a variant of the deep residual network. ResNet18-dcn introduces the concept of Deformable Convolution, which aims to improve the modeling ability of convolutional neural networks for object deformation and detail changes, so that the network can better adapt to the deformation of objects and improve the recognition performance of complex scenes and objects [31]. ResNet18-ds improves the residual structure of ResNet18 by introducing dense jump connections. By adding jump connections, ResNet18-ds can better optimize the deep network, accelerate training convergence, and improve the representation ability of the model [32]. ResNet18-dsf is another improvement of ResNet18 [33], which focuses on feature selective scaling. In the traditional ResNet, the features in the residual block are fused by direct addition, while ResNet18-dsf performs weighted scaling on different feature channels by introducing a feature selective scaling mechanism. Through feature selective scaling, ResNet18-dsf can better balance the contribution of different feature channels, improve the expression ability and generalization ability of the network, so as to obtain better performance. ResNet34-dsf is an improved version of ResNet34, and its basic principle is the same as ResNet18-dsf [34]. This improvement mainly involves the weighted scaling of feature channels, the design of residual blocks and the improvement of network performance. In this paper, we use ResNet18-dcn, ResNet18-ds, ResNet18-dsf, ResNet34-dsf and YOLOX as part of the comparative experiment. The results of the comparative experiment are shown in Table 4.

**Table 4:** RESULTS OF COMPARISON EXPERIMENTS.

Methods	Ps(%)	In(%)	Rs(%)	Cr(%)	Sc(%)	Pa(%)	mAP(%)	FPS
ResNet18-dcn	82.4	79.8	60.6	23.8	90.0	92.2	71.5	70
ResNet18-ds	84.9	84.3	63.5	45.3	94.7	92.6	77.6	70
ResNet18-dsf	85.8	83.9	61.9	54.2	97.8	92.4	78.3	51
ResNet34-dsf	97.0	84.9	64.4	53.7	95.9	93.8	80.0	64
YOLOX	94.3	88.3	57.6	36.7	92.8	82.2	75.3	60
OURS	86.3	**89.2**	**64.7**	47.3	88.5	**95.3**	78.6	**77**

Based on the experimental data in Table 3, the recognition speed of the improved CFM-YOLOv5 on the NEU-DET defect data set reaches 77 FPS. Compared with other algorithms, this method can fully meet the real-time requirements. The data in Table 4 show the comparison results of the recognition accuracy of the six defect types. The mAP of the proposed method reaches 0.786, which is similar to the accuracy of other representative algorithms. In addition, the mAP values of Inclusion, Rolled-in scale and Patches are better than other algorithms, which confirms that the effect of adding a small target prediction head proposed in this paper is more prominent for small target defect detection. The experimental results show that the optimized model can greatly improve the accuracy of target recognition, especially the recognition of small targets, while maintaining real-time performance.

Table 5 compares the CFM-YOLOv5 algorithm proposed in this paper with other mainstream target detection algorithms with various types of defect detection accuracy, overall mAP and FPS as evaluation indicators. The improved CFM-YOLOv5 model has a higher mAP value than v3s, v4s, v5s, Faster R-CNN and EDNN. In terms of FPS, the CFM-YOLOv5 model is faster than Faster R-CNN and slower than other models to varying degrees. Although the detection accuracy index is not very good, it can meet the requirements of real-time detection in industry. As shown in Table 3, the EVC module integrated in this paper and the increase of small target detection head have played a positive role in improving the accuracy of the model, and have a very obvious effect on defect detection in industrial scenes with complex background and large differences in target specifications.

**Table 5:** Different network models for metal surface defect detection performance.

Type	YOLOv3s	YOLOv4s	YOLOv5s	Faster R-CNN	EDDN	OURS
Crazing	0.369	0.445	0.403	0.376	0.417	**0.473**
Inclusion	0.579	0.732	0.862	0.802	0.620	**0.892**
Pathces	0.782	0.875	0.944	0.853	0.863	**0.953**
Pitted Surface	0.330	0.752	0.832	0.815	0.851	**0.863**
Rolled-in Scale	0.340	0.480	0.530	0.540	0.581	**0.647**
Scrathes	0.571	0.566	0.881	0.892	0.856	0.885
mAP	0.496	0.592	0.682	0.742	0.724	**0.786**
FPS	61.2	58.2	52.5	24.0	35.1	**0.770**

## Conclusion

In this paper, an improved CFM-YOLOv5 algorithm is proposed to solve the problem of metal surface defect accuracy. Through the research based on the traditional YOLOv5 s, the multi-head self-attention module of the standard transformer encoder is replaced by the EVC module, which is composed of two parallel connection blocks, and the lightweight MLP is used to capture the global information. At the same time, in order to retain the local information, the learnable visual center mechanism (LVC) is used to aggregate the local region features in the layer, which can not only extract the global long-distance correlation, but also retain the local information of the input image to the greatest extent, which is very important for the large scale of the defect data set. In the prediction part, a small target detection head is added, so that the YOLOv5 as a whole contains four prediction heads, which can capture the small defect target while capturing the normal specification target, and increase the defect recognition accuracy and robustness. After training the NEU-DET dataset, ablation experiments and analogy experiments, the mAP of CFM-YOLOv5 reaches 0.786, which is better than the existing YOLOv5 improvement method. At the same time, the FPS is maintained at 77, which can ensure the detection accuracy while ensuring the detection speed. It has a very obvious effect on defect detection in industrial scenes with complex backgrounds and large differences in target specifications. Based on the methods and conclusions proposed in this paper, we hope to help more developers and researchers in the detection of metal surface defects.
